# *In vitro* hepatic aflatoxicol production is related to a higher resistance to aflatoxin B_**1**_ in poultry

**DOI:** 10.1038/s41598-020-62415-y

**Published:** 2020-03-26

**Authors:** Hansen W. Murcia, Gonzalo J. Diaz

**Affiliations:** 1 Instituto de Biotecnología, Universidad Nacional de Colombia, Bogotá, D.C. 111321 Colombia; 2 Laboratorio de Toxicología, Facultad de Medicina Veterinaria y de Zootecnia, Universidad Nacional de Colombia, Bogotá, D.C. 111321 Colombia

**Keywords:** Oxidoreductases, Oxidoreductases

## Abstract

A study was conducted to determine the cytosolic *in vitro* hepatic enzymatic kinetic parameters V_*max*_, K_*M*_, and intrinsic clearance (CL_*int*_) for aflatoxin B_1_ (AFB_1_) reductase [aflatoxicol (AFL) production] and AFL dehydrogenase (AFB_1_ production) in four commercial poultry species (chicken, quail, turkey and duck). Large differences were found in AFB_1_ reductase activity, being the chicken the most efficient producer of AFL (highest CL_*int*_ value). Oxidation of AFL to AFB_1_ showed only slight differences among the different poultry species. On average all species produced AFB_1_ from AFL at a similar rate, except for the turkey which produced AFB_1_ from AFL at a significantly lower rate than chickens and quail, but not ducks. Although the turkey and duck showed differences in AFL oxidation V_*max*_ and K_*M*_ parameters, their CL_*int*_ values did not differ significantly. The ratio AFB_1_ reductase/AFL dehydrogenase enzyme activity was inversely related to the known *in vivo* sensitivity to AFB_1_ being highest for the chicken, lowest for the duck and intermediate for turkeys and quail. Since there is no evidence that AFL is a toxic metabolite of AFB_1_, these results suggest that AFL production is a detoxication reaction in poultry. Conversion of AFB_1_ to AFL prevents the formation of the AFB_1_-8,9-*exo*-epoxide which, upon conversion to AFB_1_-dihydrodiol, is considered to be the metabolite responsible for the acute toxic effects of AFB_1_.

## Introduction

**Figure 1:**
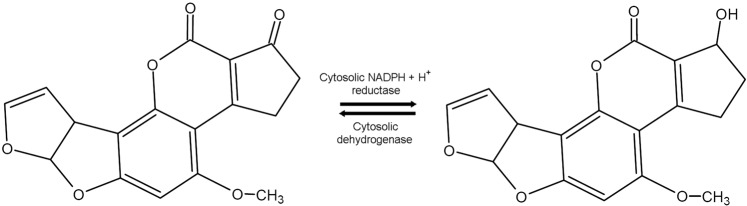
Enzymatic reduction of aflatoxin B_1_ into aflatoxicol by aflatoxin B_1_ cytosolic NADPH + H^+^ reductase and oxidation of aflatoxicol into AFB_1_ by aflatoxicol cytosolic dehydrogenase.

Aflatoxicol (AFL) is a metabolite produced by the enzymatic reduction of carbon 1 (C1) in the cyclopentanone ring of aflatoxin B_1_ (AFB_1_). This compound was identified for the first time as a product of AFB_1_ of the non-aflatoxigenic strain NRRL 2575 of *Dactylium dendroides*^1,2^. After its discovery it was found that the enzymatic reduction of AFB_1_ to AFL, as well the enzymatic oxidation of AFL back to AFB_1_ (Fig. 1), occur in animal species such as duck, turkey, chicken, rabbit, Guinea-pig, mouse, rat^3,4^ and rainbow trout (*Oncorhynchus mykiss*)^5^. Additionally, AFL oxidation to AFB_1_ has been reported in primary rat hepatocyte culture^6^, whereas AFB_1_ reduction to AFL has been found in rat erythrocyte cytosol^7^.

In AFB_1_ sensitive species like the rainbow trout or rabbit (LD_50_ value of 0.81 and 0.30 mg AFB_1_/kg body weight, respectively^8^) hepatic *in vitro* incubations biotransform around 60% of an initial concentration of AFB_1_ into AFL^9^. However, in hepatic *in vitro* incubations with the most sensitive poultry species (the duck) it has been found that only about 10% of the initial concentration of AFB_1_ is converted to AFL^10^; no evidence of this reaction was seen in rat, mouse, Rhesus monkey or humans. Bailey *et al*.^11^ proposed that AFL could be a reservoir of AFB_1_ in sensitive species like duck or rainbow trout. If AFL is a storage form of AFB_1_, the half-life of the toxin would be longer and could potentially lead to chronic effects^4,12^. In fact, the hypothesis that AFL may be a detoxification product if conjugated with glucuronic acid has been suggested previously^13^. Due to the high sensitivity of rainbow trout to AFL, it was proposed that AFL could also generate DNA adducts like those produced by AFB_1_^11^; however, there is no proof that this reaction actually occurs *in vivo*. AFL epoxidation has only been achieved by using chemical oxidizing agents like dimethyldioxirane or *m*-chloroperbenzoic acid^11,14^. On the other hand, if AFB_1_ is poorly biotransformed into AFL, it will be available for further biotransformation into aflatoxin B_1_-8,9-*exo*-epoxide (AFBO) by cytochrome P450 (CYP) enzymes, generating DNA adducts^12,15^.

Information about the enzymatic reduction of AFB_1_ to AFL and the oxidation of AFL back to AFB_1_ in poultry species is scarce. Lozano and Diaz^16^ reported that hepatic cytosolic *in vitro* reduction rates of AFB_1_ follow the order turkey > duck > quail > chicken, but did not determine enzymatic parameters such as maximal velocity (V_*max*_), Michaleis-Menten constant (K_*M*_) or intrinsic clearance (CL_*int*_). Since it is well-known that different poultry species exhibit different sensitivities to AFB_1_, the present study was conducted with the aim of investigating possible differences in the enzymatic kinetic parameters K_*M*_, V_*max*_ and CL_*int*_ for AFB_1_ reduction and AFL oxidation and to relate these differences with the known *in vivo* susceptibility to AFB_1_ of each of these four poultry species.

## Results

**Figure 2:**
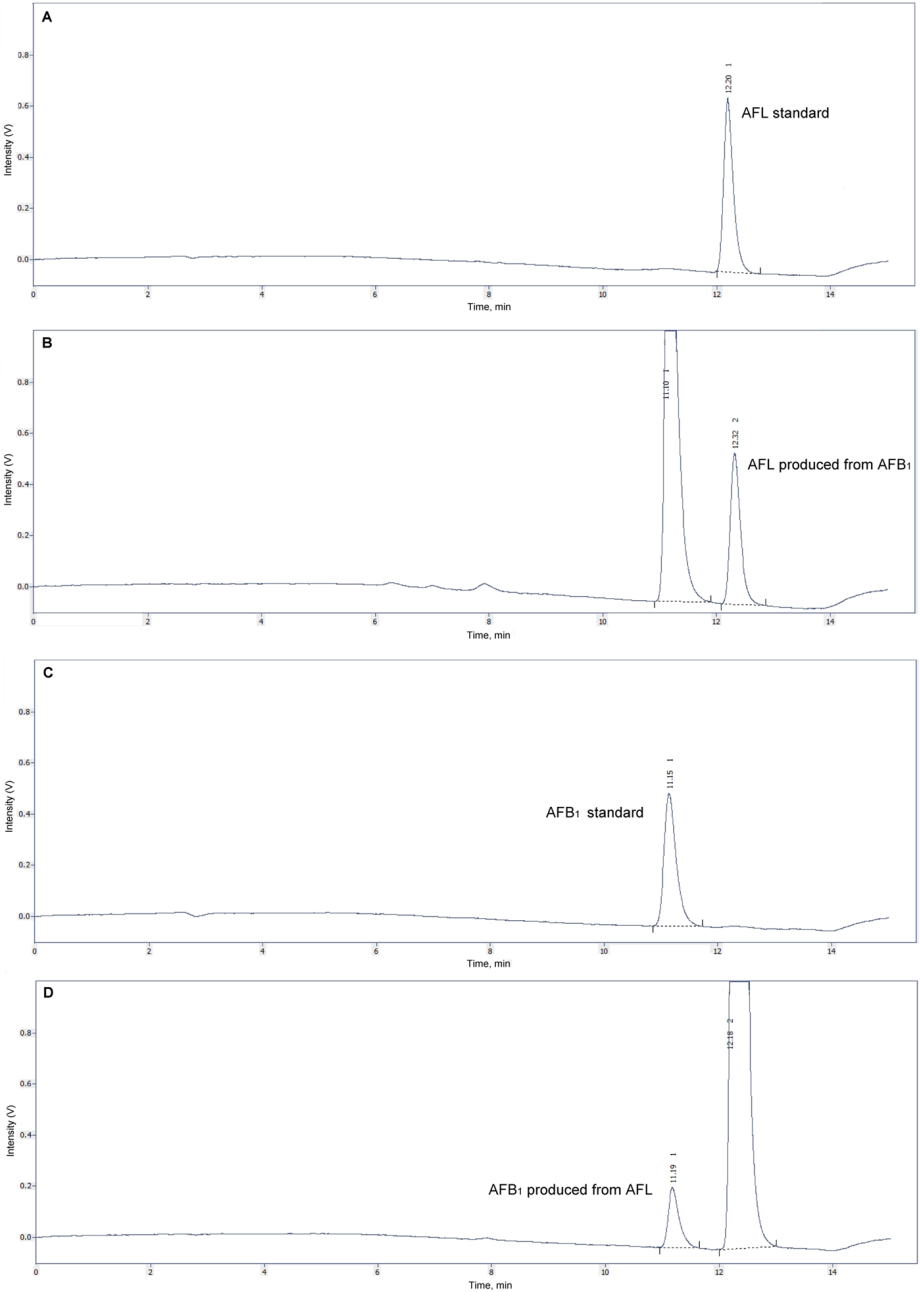
Chromatograms of aflatoxin B_1_ reductase activity (**A**,**B**) and aflatoxicol dehydrogenase activity (**C**,**D**) products. (**A**) Aflatoxicol standard (t_*R*_ = 12.20, 318.4 fmol in colum). (**B**) Enzymatic production of aflatoxicol (t_*R*_ = 12.32, 334.3 fmol in column) from aflatoxin B_1_ (t_*R*_ = 11.10, 36.7 *μ*M in incubation). (**C**) Aflatoxin B_1_ standard (t_*R*_ = 11.15, 1.28 pmol in column). (**D**) Enzymatic production of aflatoxin B_1_ (t_*R*_ = 11.19, 542.4 fmol in colum) from aflatoxicol (t_*R*_ = 12.18, 36.5 *μ*M in incubation).

**Figure 3:**
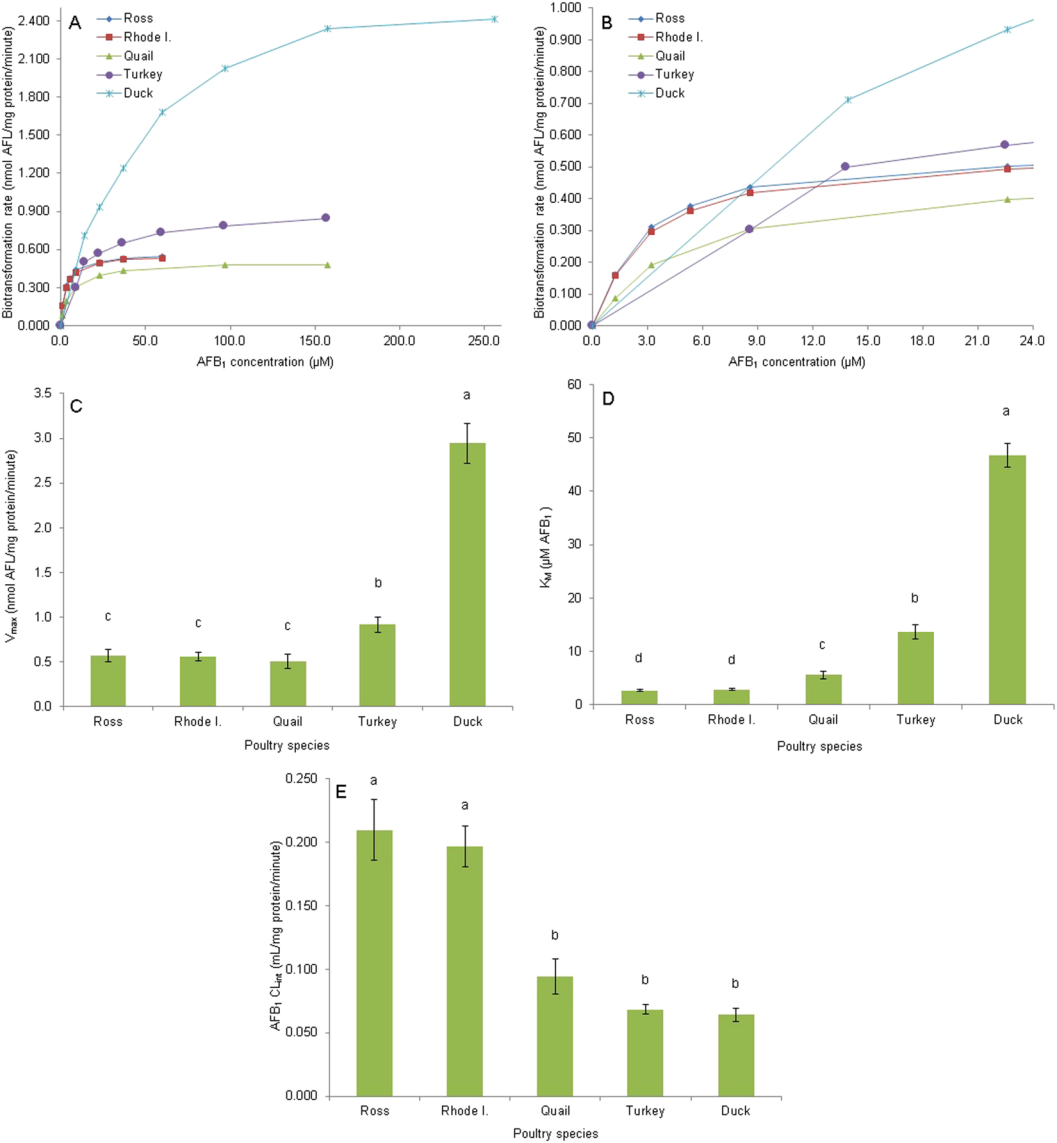
Enzyme kinetic parameters of cytosolic *in vitro* aflatoxicol production from AFB_1_. (**A**) Saturation curves at AFB_1_ concentrations of 1.23 to 59.7 *μ*M for chicken breeds, 1.23 to 157 *μ*M for quail and turkey and 13.9 to 256 *μ*M for duck. (**B**) Saturation curves in the AFB_1_ concentration range from 0 to 24 *μ*M. (**C**) Maximal velocity (V_*max*_). (**D**) Michaelis-Menten constant (K_*M*_). (**E**) Intrinsic clearance (CL_*int*_; V_*max*_/K_*M*_). Species mean values with the same letter do not differ significantly. Statistical differences (P < 0.05) were calculated using the Kruskal-Wallis test. Values are means ± SEM (**C–E**) of 12 birds.

Enzymatic products of AFB_1_ reductase and AFL dehydrogenase activities are presented in Fig. 2. Figure 2A presents the chromatogram obtained from the AFL standard used to confirm the AFL enzymatic production. (Figure 2B) show the chromatogram where AFB_1_ was used as substrate. Figure 2C presents the chromatogram where AFB_1_ standard is used to confirm the AFB_1_ enzymatic production and (Fig. 2D) presents the chromatogram where AFL was used as substrate. The saturation curves of enzymatic reduction of AFB_1_ to AFL as well as the enzymatic parameters V_*max*_, K_*M*_ and CL_*int*_ for the four poultry species studied are presented in Fig. 3. The biotransformation rate of AFB_1_ into AFL was highest in the duck, which seems to saturate at a concentration of 256 *μ*M of AFB_1_. On the other hand, both turkey and quail reach maximal velocity at 157 *μ*M, and the chicken breeds at 59.7 *μ*M (Fig. 3A). Figure 3B presents the same saturation curves in the 0 to 24 *μ*M AFB_1_ concentration range, where it is observed that the chicken breeds produce more AFL at AFB_1_ concentrations below 9 *μ*M compared with the other poultry species. In all cases adjustment of the dataset to the Michaelis Menten equation resulted in coefficients of determination (R^2^) ≥ 0.99. Figure 3C shows the results for the V_*max*_ AFL production where the duck had the highest value (2.94 ± 0.78 nmol AFL/mg protein/minute), which was 3.2 times higher than the one found for the turkey (0.92 ± 0.29 nmol AFL/mg protein/minute). There were no differences between the chicken breeds (0.57 ± 0.24 and 0.56 ± 0.18 nmol AFL/mg protein/minute for the Ross and the Rhode Island Red, respectively) and quail (0.50 ± 0.27 nmol AFL/mg protein/minute). Only the Ross breed presented significant differences for V_*max*_ between sexes (0.72 ± 0.21 and 0.42 ± 0.18 nmol AFL/mg protein/minute for males and females respectively). K_*M*_ values for AFB_1_ reduction also showed significant differences (Fig. 3D), with the duck showing the highest value (46.8 ± 7.7 *μ*M of AFB_1_), followed by the turkey (13.6 ± 4.5 *μ*M of AFB_1_) and the quail (5.6 ± 2.6 *μ*M of AFB_1_). No differences were found between the chicken breeds (2.7 ± 0.7 and 2.9 ± 0.6 *μ*M of AFB_1_ for Ross and Rhode Island Red, respectively). No differences by sex for this parameter were found for any of the poultry species studied. Measurement of the enzymatic efficiency of AFL production as CL_*int*_ (Fig. 3E) showed that chicken breeds have the most efficient enzymatic AFL production system, with values for Ross and Rhode Island Red breeds of 0.21 ± 0.08 and 0.20 ± 0.056 mL/mg protein/minute, respectively. Quail and turkey showed an intermediate efficiency with values of 0.095 ± 0.05 and 0.068 ± 0.01 mL/mg protein/minute respectively, while the duck had the lowest value among the poultry species evaluated (0.064 ± 0.02 mL/mg protein/minute). In regard to sex, only quail showed significant differences between sexes (0.12 ± 0.05 and 0.07 ± 0.03 mL/mg protein/minute for males and females respectively).

**Figure 4:**
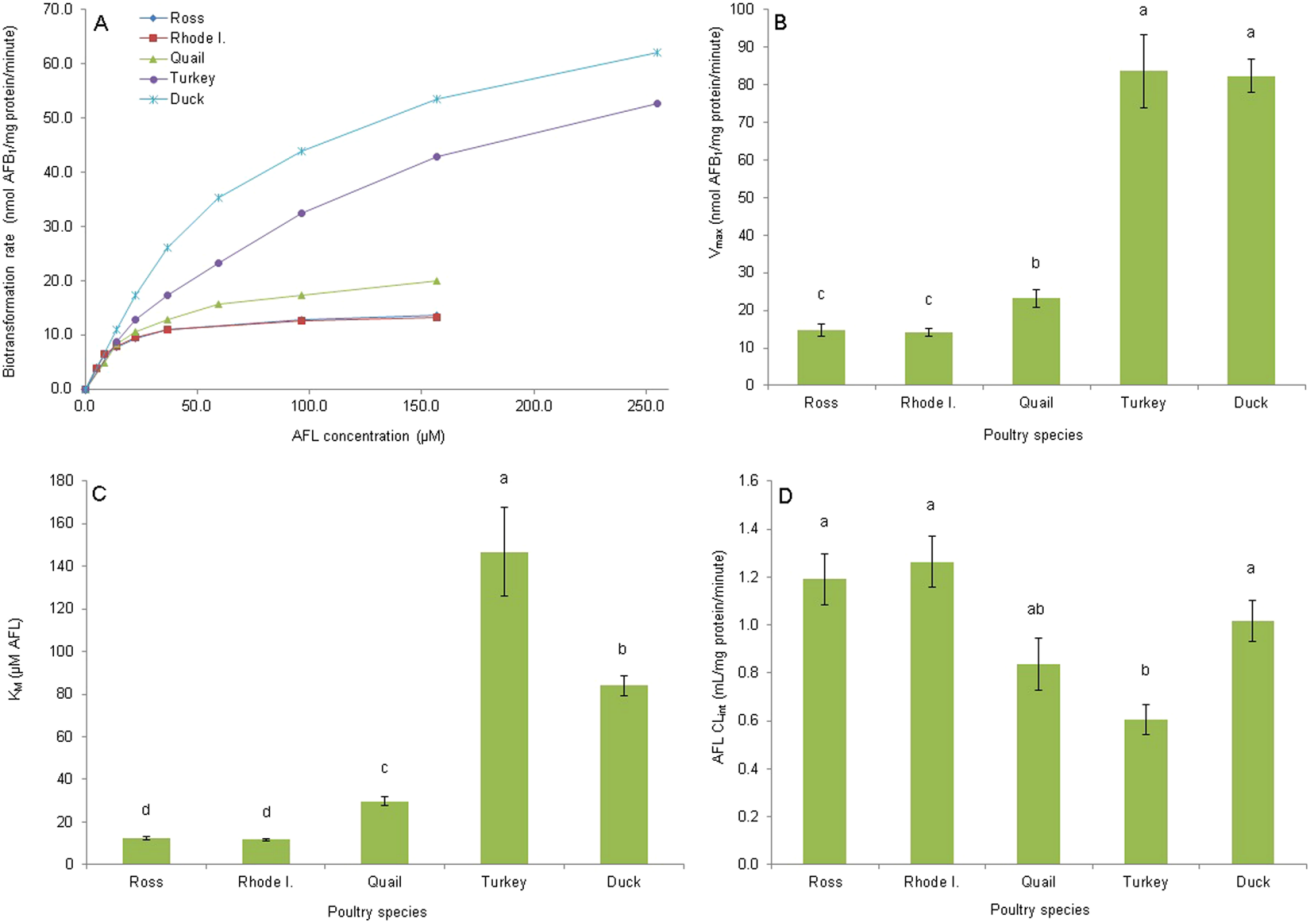
Enzyme kinetic parameters of cytosolic *in vitro* AFB_1_ production from aflatoxicol (AFL). (**A**) Saturation curve at AFL concentrations of 5.2 to 156.7 *μ*M for chicken breeds and quail, and of 13.8 to 254.7 *μ*M for turkey and duck species. (**B**) Maximal velocity (V_*max*_). (**C**) Michaelis-Menten constant (K_*M*_). (**D**) Intrinsic Clearance (CL_*int*_; V_*max*_/K_*M*_). Species mean values with the same letter do not differ significantly. Statistical differences (P < 0.05) were calculated using the Kruskal-Wallis test. Values are means ± SEM (**B**–**D**) of 12 birds.

  Figure 4 shows the saturation curves, as well as V_*max*_, K_*M*_ and CL_*int*_ values for the enzymatic oxidation of AFL to AFB_1_. For this reaction both turkey and duck cytosolic enzymes seem not to be completely saturated at a concentration of 254.7 *μ*M AFL; however, the other species saturated at 156.7 *μ*M (Fig. 4A). V_*max*_ was highest in the turkey and the duck, with values of 83.6 ± 33.9 and 82.3 ± 15.4 nmol AFB_1_/mg protein/minute respectively, followed by the quail (23.2 ± 7.9 nmol AFB_1_/mg protein/minute) and the chicken breeds (14.7 ± 5.6 and 14.2 ± 3.5 nmol AFB_1_/mg protein/minute for Ross and Rhode Island Red chickens, respectively; Fig. 4B). Only the Ross chicken breed presented significant differences between sexes (18.5 ± 4.9 and 10.9 ± 3.4 nmol AFB_1_/mg protein/minute for males and females respectively). K_*M*_ was lowest for the chicken breeds with values of 12.3 ± 2.8 and 11.6 ± 2.3 *μ*M of AFL for Ross and Rhode Island Red chickens, respectively. Quail presented a higher value (29.8 ± 6.8 *μ*M of AFL), which was even higher for the duck (84.0 ± 16.5 *μ*M of AFL), and highest for the turkey (146.8 ± 72.4 *μ*M of AFL; Fig. 4C). Significant differences between sexes were observed for Ross chickens (14.2 ± 2.76 and 10.4 ± 1.15 *μ*M of AFL for males and females respectively), quail (24.6 ± 3.6 and 34.9 ± 5.1 *μ*M of AFL for males and females respectively) and turkeys (105.7 ± 30.6 and 187.9 ± 80.9 *μ*M of AFL for males and females respectively). Only the turkey CL_*int*_ value (0.6 ± 0.2 mL/mg protein/minute) differed significantly from the chicken breeds and duck (1.2 ± 0.4, 1.3 ± 0.4 and 1.0 ± 0.3 mL/mg protein/min, respectively; Fig. 4D). Quail CL_*int*_ value did not differed from any of the other species and it was the only species that showed significant differences between sexes for this parameter (1.1 ± 0.3 and 0.6 ± 0.2 mL/mg protein/minute for males and females respectively).

Comparisons of the ratio AFB_1_ reductase CL_*int*_/AFL dehydrogenase CL_*int*_ showed significant differences between species, where Ross (0.175 ± 0.048) and Rhode Island Red chicken breeds (0.159 ± 0.025) showed the highest and similar values, followed by quail (0.114 ± 0.020) and turkey (0.124 ± 0.038) which also presented similar values, and finally the duck (0.064 ± 0.017) with the lowest value.

## Discussion

The *in vivo* sensitivity to AFB_1_ in poultry species (duck > turkey > quail > chicken) has been proposed to be related to qualitative and/or quantitative differences in AFB_1_ metabolism^17^. Hepatic cytosolic enzymes of poultry species can reduce AFB_1_ to AFL^16^ and the inverse reaction is also possible^3,4^. In the present study, determination of the enzymatic kinetic enzymatic parameters for these reactions revealed that resistant species (e.g. chickens, quail) reach V_*max*_ for AFL production at lower AFB_1_ concentrations, as determined by their lower K_*M*_ values. On the other hand, turkeys and ducks show higher K_*M*_ values associated with CL_*int*_ values three times lower than those of the chicken, suggesting a lower enzymatic efficiency in converting AFB_1_ to AFL. Therefore, less sensitive species produce AFL more efficiently than more sensitive ones, at low AFB_1_ concentrations. Although in resistant species like the chicken breeds the V_*max*_ value was about six times lower that in sensible species like the duck or turkey, the chickens reach V_*max*_ at lower concentrations of AFB_1_ than the other species. This fact can be very significant since the AFB_1_ concentrations expected to occur in the hepatocyte upon AFB_1_ exposure are in the nanomolar or even the femtomolar order^18^. On the other hand, although large differences were found for AFB_1_ reduction, dehydrogenation of AFL back to AFB_1_ showed only minor differences among the different poultry species. On average all species produced AFB_1_ from AFL at a similar rate, except for the turkey.

In order to elucidate if the proposal of Bailey *et al*.^11^ that AFL could be a reservoir of AFB_1_ in sensitive species, the ratio “AFB_1_ reductase activity CL_*int*_/AFL dehydrogenase activity CL_*int*_” was calculated. Surprisingly, this ratio was highest for the chickens (0.18 and 0.16), intermediate in quail and turkey (0.11) and lowest (0.06) for the most sensitive species (duck). These results suggest that the amount of AFL produced from AFB_1_ (or AFL accumulation) follows the order Ross chicken = Rhode Island Red chicken > quail = turkey > duck. Interestingly, this is the opposite order to AFB_1_*in vivo* sensitivity in which the duck is the most sensitive, the chicken is the less sensitive and the quail and turkey are intermediate in sensitivity^17^. In other sensitive species like the rainbow trout (*Oncorhynchus mykiss*) the AFB_1_ production rate from AFL is lower than the AFL production from AFB_1_^12,15^ and therefore it has been suggested that the ratio AFB_1_ reductase/AFL dehydrogenase is higher in species prone to develop acute aflatoxicosis^19^. The results obtained in the present trial clearly show that this contention is not valid for the poultry species studied. A possible explanation for this discrepancy could be that in poultry species production of AFL represents a way to avoid epoxidation of AFB_1_ to AFBO, and the subsequent formation of its hydrolysis product aflatoxin B_1_ dihydrodiol (AFB_1_-dhd), the proposed acutely toxic metabolite of AFB_1_^20^. It is important to highlight that duck liver enzymes can oxidize AFB_1_ to AFBO more efficiently at lower AFB_1_ concentrations than turkeys, chickens, or quail^20^. If the duck cannot biotransform AFB_1_ to AFL efficiently, AFB_1_ will be available for bioactivation, producing AFBO through CYP enzymes. In the case of the chicken, oxidation of AFB_1_ to AFBO is less efficient than in other species^20^, and the reduction of AFB_1_ to AFL will further reduce the AFB_1_ available to be epoxidated. The subcellular localization of the AFB_1_ reductase (cytosol) and CYP enzymes (smooth endoplasmic reticulum) requires that the toxin comes first in contact with the reductase and if this activity is highly efficient there will be no toxin available for bioactivation by CYP enzymes. It is possible that this is the strategy used by the chicken in order to be able to tolerate AFB_1_ doses that could kill sensitive species. In fact, production of AFL by the chicken liver could be considered a true detoxication pathway since there is no evidence of AFL enzymatic epoxidation through CYP enzymes, despite the presence of the 8,9-double bond in the furan ring of the molecule. Research conducted to determine the mechanism of toxicity of AFL failed to find any evidence of adduction of AFL with DNA^12,15^ and it was suggested that the mechanism of adduction from AFL is due to its oxidation to AFB_1_ and the subsequent epoxidation of AFB_1_ through CYP enzymes to AFBO. According to this, AFL is not toxic *per se* and storage of AFL will not generate toxic effects. Research conducted in our laboratory failed to detect any glucuronidation or sulfoconjugation of AFL in the same poultry species studied. It might be possible that AFL is a substrate of a hepatocyte transmembrane transport^21^ that removes the metabolite from the hepatocytes, thereby finalizing the detoxication pathway.

In summary, the present study reports for the first time the enzymatic kinetic parameters of AFB_1_ reduction and AFL oxidation in poultry species. The ratio AFB_1_ reduction/AFL oxidation was found to be inversely related to the known *in vivo* susceptibility to AFB_1_. Further, the most resistant species (chicken) was found to be the most efficient producer of AFL. It is important to note that the chicken is so resistant to AFB_1_ that it not only tolerates dietary concentrations that could acutely kill other animals, but also grows better when AFB_1_ in present in its diet^22^. Although some studies report adverse effects on different clinical or performance parameters, these results need to be analyzed with caution since they need to be contextualized within the expected levels of contamination in feed ingredients and complete feeds^23^. Our results, therefore, contradict the common theory that AFL acts as a reservoir of AFB_1_, thereby increasing its toxicity. On the other hand, a recent study has shown that sensitive species produce more AFB_1_-dhd (from AFBO) than resistant ones^20^ and we have now found that the latter species also produce more AFL. Low AFBO production coupled with high AFL production from AFB_1_ could be the explanation for the high resistance of chickens and other resistant poultry to AFB_1_. However, more research is needed to determine the metabolic fate of the AFB_1_-dhd and AFL and the enzymatic kinetic parameters of AFBO-glutathione conjugation.

## Methods

### Reagents

Glucose 6-phosphate sodium salt, nicotinamide dinucleotide phosphate (NADP^+^), glucose 6-phosphate dehydrogenase, ethylenediaminetetraacetic acid (EDTA), bicinchoninic acid solution (sodium carbonate, sodium tartrate, sodium bicarbonate and sodium hydroxide 0.1 N, pH 11.25), copper sulphate pentahydrate, formic acid, dimethylsulfoxide (DMSO), sucrose, glycerol, and bovine serum albumin were from Sigma-Aldrich (St. Louis, MO, USA). Aflatoxicol and aflatoxin B_1_ were purchased from Fermentek Ltd. (Jerusalem, Israel). Sodium chloride and magnesium chloride pentahydrate were purchased from Mallinckrodt Baker (Phillipsburg, NJ, USA). Sodium phosphate monobasic monohydrate and sodium phosphate dibasic anhydrous were from Merck (Darmstadt, Germany). Methanol, acetonitrile and water were all HPLC grade.

### Cytosolic fraction processing

Liver fractions were obtained from 12 healthy birds (6 males and 6 females) from each of the following species and ages: seven-week old Ross and Rhode Island Red chickens (*Gallus gallus ssp. domesticus*), eight-week old turkeys (*Meleagris gallopavo*), eight-week old quails (*Coturnix coturnix japonica*) and nine-week old Pekin ducks (*Anas platyrhynchos ssp. domesticus*). The birds were sacrificed by cervical dislocation, and their livers extracted immediately, washed with cold PBS buffer (50 mM phosphates, pH 7.4, NaCl 150 mM), cut into small pieces and stored at  −70 °C until processing. The experiment was conducted following the welfare guidelines of the Poultry Research Facility and was approved by the Bioethics Committee, Faculty of Veterinary Medicine and Zootechnics, National University of Colombia, Bogotá D.C., Colombia (approval document CB-FMVZ-UN-033-18). Frozen liver samples were allowed to thaw, and 2.5 g were minced and homogenized for 1 minute with a tissue homogenizer (Cat X120, Cat Scientific Inc., Paso Robles, CA, USA) with 10 mL of extraction buffer (phosphates 50 mM pH 7.4, EDTA 1 mM, sucrose 250 mM). The homogenates were then centrifuged at 12000 × *g* for 30 minutes at 4 °C (IEC CL31R Multispeed Centrifuge, Thermo Scientific, Waltham, MA, USA). After this first centrifugation, the supernatants (approximately 10 mL) were transferred into ultracentrifuge tubes kept at 4 °C and centrifuged for 90 minutes at 100000 × *g* (Sorval WX Ultra 100 Centrifuge, Thermo Scientific, Waltham, MA, USA). The resulting supernatants (corresponding to the cytosolic fraction) were fractioned in microcentrifuge tubes and stored at  −70 °C. An aliquot of each sample was taken to determine its protein content by using the bicinchoninic acid protein quantification method according to Redinbaugh and Turley^24^.

### Cytosolic incubations

Incubations were made per each animal at seven different substrate concentrations, with each concentration run in duplicate. For AFB_1_ reductase enzyme activity, *in vitro* incubations were carried out in 1.5 mL microcentrifuge tubes kept at 39 °C (the normal average poultry body temperature) containing 5 mM glucose 6-phosphate, 0.5 mM NADP^+^, 0.5 I.U. glucose 6-phosphate dehydrogenase, 1 *μ*L of AFB_1_ in DMSO at concentrations ranging from 1.23 to 256 *μ*M, and 100 *μ*g of cytosolic protein for chicken breeds and quail or 25 *μ*g for turkey and duck. For AFL dehydrogenase enzyme activity incubation contained 0.5 mM NADP^+^, 1 *μ*L of AFL in DMSO at concentrations ranging from 5.2 to 254.7 *μ*M and 10 *μ*g of cytosolic protein except for turkey, where 5 *μ*g were used. All volumes were completed with incubation buffer (phosphates 50 mM pH 7.4, MgCl 5 mM, EDTA 0.5 mM), and the reaction stopped after 10 minutes with 250 *μ*L of ice-cold acetonitrile. The stopped incubations were centrifuged at 15000 × *g* for 10 minutes and 2 *μ*L of a 1:10 dilution in mobile phase (except for turkey and duck, where a 1:100 dilution was made) were analyzed by high-performance liquid chromatography (HPLC) as described below.

### Chromatographic conditions (HPLC)

The production of AFL or AFB_1_ in each incubation was quantitated in a Shimadzu Prominence system (Shimadzu Scientific Instruments, Columbia, MD, USA) equipped with a DGU-20A3R degassing unit, two LC-20AD pumps, a SIL-20AC_*HT*_ autosampler, a CTO-20A column oven, an RF-20A_*XS*_ fluorescence detector, and a CBM-20A bus module, all controlled by “LC Solutions” software. The chromatography was carried out on an Alltech Alltima HP C18, 150 mm × 3.0 mm chromatographic column (Alltech Associates Inc., Deerfield, IL, USA) kept at 40 °C. The mobile phase was a linear gradient of solvent A (water - 0.1% formic acid) and B (acetonitrile:methanol, 1:1–0.1% formic acid), as follows: 0 min: 25% B, 1 min: 25% B, 10 min: 60% B, 10.01 min: 25% B, and 17 min: 25% B. The flow rate was 0.4 mL/min and the fluorescence detector was set at excitation and emission wavelengths of 365 nm and 425 nm, respectively. The in-vial concentration of AFL and AFB_1_ was quantitated using standards of AFL and AFB_1_ of known concentration. The linearity of the response for AFL was confirmed with a calibration curve for AFL with in column amounts ranging from 12.7 to 127.4 fmol, for which an R^2^ value of 0,9989 was obtained. The calibration curve for AFB_1_ quantitation corresponded to in column amounts of AFB_1_ ranging between 128–1280 fmol, with an R^2^ of 0,9998. Analytical method precision was estimated by the Relative Standard Deviation (RSD) of the results obtained for determinations of AFL and AFB_1_ at the intermediate level of the calibration curves in triplicate. RSD values for AFL and AFB_1_ were 2 and 3%, respectively. Recovery was estimated at 100% since the concentration of the analytes AFL and AFB_1_ found in blank incubations corresponded to the amount expected from the calculation based on the external standard calibration curves. This result was expected since the matrix corresponded to incubation buffer that was not subjected to any type of extraction or clarification procedures.

### Statistical analysis

The enzymatic parameters K_*M*_ and V_*max*_ were determined by non-linear regression using the Marquardt method adjusting the data to the Michaelis-Menten enzyme kinetics using the equation: v = V_*max*_[S]/K_*M*_ + [S], where v is the enzyme reaction velocity, [S] represents substrate concentration, V_*max*_ represents maximal velocity and K_*M*_ represents the Michaelis-Menten constant. Intrinsic clearance (CL_*int*_) was calculated as the ratio V_*max*_/K_*M*_. Inter-species differences in enzymatic kinetic parameters were determined by using the Kruskal-Wallis test, while nonparametric multiple comparisons were made by using the Dwass-Steel-Critchlow-Fligner method. All analyses were performed using the Statistical Analysis System software^25^.
